# Activation of oxidative carbon metabolism by nutritional enrichment by photosynthesis and exogenous organic compounds in the red alga *Cyanidioschyzon merolae*: evidence for heterotrophic growth

**DOI:** 10.1186/s40064-015-1365-0

**Published:** 2015-09-28

**Authors:** Takashi Moriyama, Natsumi Mori, Naoki Sato

**Affiliations:** Department of Life Sciences, Graduate School of Arts and Sciences, The University of Tokyo, Komaba 3-8-1, Meguro-ku, Tokyo, 153-8902 Japan; JST, CREST, K’s Gobancho 7 Gobancho, Chiyoda-ku, Tokyo, 102-0076 Japan

**Keywords:** Alga, Glycolysis, Heterotrophic growth, Obligate-photoautotrophic growth, Organic nutrition, Photosynthesis, Respiration

## Abstract

Respiration is an important process in photosynthetic organisms, as it is in other organisms, for the supply of ATP and metabolites required for biosynthesis. Furthermore, individual enzymatic activity is subject to regulation by metabolic intermediates in glycolysis and the citric acid cycle. However, little is known about how glycolysis or catabolism are related to photosynthetic activity or accumulation of photosynthetic products. We previously developed a flat-plate culture apparatus assembled from materials commonly used for gel electrophoresis, which enables high-density culture of the unicellular red alga *Cyanidioschyzon merolae*. In this study, a stationary dense culture of *C*. *merolae*, when re-activated in this culture apparatus, exhibited an accumulation of photosynthetically produced starch. We demonstrated that respiratory activity increased during the culture period, while photosynthetic activity remained constant. Gene expression analysis revealed that the genes involved in cytosolic glycolysis and the citric acid cycle were selectively activated, compared to the genes for the oxidative pentose phosphate pathway and the Calvin–Benson cycle. Measurements of the respiratory rate after addition of various organic substances showed that *C*. *merolae* can utilize almost any exogenous organic compound as a respiratory substrate, although the effectiveness of each compound was dependent on the culture time in the flat-plate culture, suggesting that glycolysis was rate-limiting to respiration, and its activity depended on the level of photosynthetic products within the cells. We also demonstrated that organic substances increased the rate of cell growth under dim light and, interestingly, *C*. *merolae* could grow heterotrophically in the presence of glycerol. Obligate photoautotrophy should be considered an ecological, rather than physiological, characteristic of *C*. *merolae*.

**Electronic supplementary material:**

The online version of this article (doi:10.1186/s40064-015-1365-0) contains supplementary material, which is available to authorized users.

## Background

Photosynthetic organisms consume oxygen through respiration and produce it through photosynthetic oxygen evolution. In plants, the respiratory rate is estimated to be 1/6 to 1/20 of the photosynthetic rate (Radoglou and Teskey 1997). Respiration is the main source of cytosolic ATP, even in light-irradiated leaves (Krömer 1995), and also supplies metabolites required for biosynthesis, such as 2-oxoglutarate (2OG, Hoefnagel et al. 1998). Defects in a gene that encodes part of the respiratory chain lead to poor leaf growth and a decrease in the rate of photosynthesis (Vedel et al. 1999). Plant respiration is regulated by phosphoenolpyruvate (PEP)-induced inhibition of ATP-dependent phosphofructokinase (PFK) and inverse activation of PFK by inorganic phosphate (Plaxton 1996). In addition, an increase in NADH concentration represses the activity of dehydrogenases in the citric acid cycle (McIntosh and Oliver 1992). In the green alga *Chlorella pyrenoidosa*, the rate of respiration is positively correlated with growth rate and gradually decreases in darkness (Geider and Osborne 1989). Although regulation of the enzymatic reactions in glycolysis and the citric acid cycle has been observed, little is known about how respiratory activity relates to photosynthetic activity or to the accumulation of photosynthetic products.

The unicellular red alga *Cyanidioschyzon merolae* thrives in hot (up to 50 °C) and acidic (pH 1.5–2.5) springs, while maintaining an intracellular pH of 6.4 (Zenvirth et al. 1985). The cell structure of *C*. *merolae* is simple, consisting of one plastid and one mitochondrion per cell. The nuclear (Matsuzaki et al. 2004; Nozaki et al. 2007), mitochondrial (Ohta et al. 1998), and plastid (Ohta et al. 2003) DNA have been sequenced. The nuclear genome of *C*. *merolae* is remarkably compact in structure: its size is 16.5 Mbp; its predicted number of protein-coding genes is 4774 (Nozaki et al. 2007; Moriyama et al. 2014a), with low redundancy; and its number of intron-containing genes is 26. Transformation of *C*. *merolae* is easily achieved using the polyethylene glycol (PEG) method (Ohnuma et al. 2008), and subcellular localization of 3 × hemagglutinin (HA)-tagged and GFP-fused proteins in transformants has been reported (Ohnuma et al. 2008; Watanabe et al. 2011; Moriyama et al. 2014b). Comprehensive subcellular localization analysis has shown that the subcellular distribution of carbon metabolic pathways in *C*. *merolae* is essentially identical to that in *Arabidopsis thaliana*; namely, glycolysis is localized to the cytosol and plastid, while the Calvin–Benson and citric acid cycles exist in the plastid and mitochondrion (Moriyama et al. 2014a). However, *C*. *merolae* lacks transaldolase, an enzyme of the oxidative pentose phosphate pathway.

*Cyanidioschyzon merolae* belongs to the order Cyanidiales in the phylum Rhodophyta. This order consists of two genera besides *Cyanidioschyzon*: *Galdieria* and *Cyanidium*. *Galdieria sulphuraria* (Gross and Schnarrenberger 1995) and *Cyanidium caldarium* (Rigano et al. 1977) both grow under heterotrophic conditions, whereas *C*. *merolae* is thought to be an obligate photoautotroph (Raven et al. 1990). Genomic analyses of genes related to carbon metabolism have suggested that *C*. *merolae*, like *G*. *sulphuraria*, harbors metabolic pathways for floridoside, trehalose, storage glucans, and matrix polysaccharides (Barbier et al. 2005). For storage glucans, *C*. *merolae* contains semiamylopectin-type glucans without amylose (Hirabaru et al. 2010). *C*. *merolae* is predicted to contain a minimal set of metabolic transporters, such as those for triose phosphate, but no aquaporin-type glycerol permease for uptake of glycerol from the environment, or plastidic dicarboxylate translocators, which are required for nitrogen assimilation and for photorespiration and which are conserved in green plants and algae (Barbier et al. 2005; Tyra et al. 2007).

In this study, we reexamined the utilization efficiency of exogenous organic substances as a carbon source in *C*. *merolae*, which previously was thought to be an obligate photoautotroph and to not have any transporters for uptake of exogenous compounds. Our results indicate that *C*. *merolae* efficiently utilizes various exogenous substrates for respiration and growth, and *C*. *merolae* can heterotrophically grow in glycerol-containing medium, but not in medium containing glucose, succinic acid, or lactic acid. We also examined the regulation of respiration by exogenously added organic compounds in cells as a function of flat-plate culture time. Our results suggest that the glycolytic pathway is rate-limiting to respiration, and the activity of the pathway varies dramatically depending on the amount of photosynthetic products.

## Results

### Physiological changes in *C*. *merolae* cells grown in flat-plate culture

**Fig. 1:**
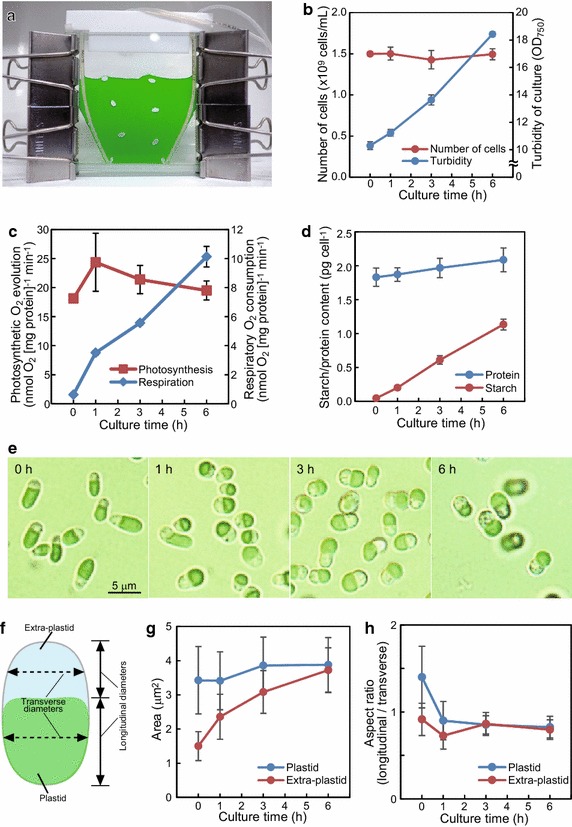
Physiological changes in *C. merolae* cells grown in the flat-plate culture apparatus. **a** A picture of the flat-plate culture. Changes in number of cells and turbidity of culture (**b**), photosynthetic and respiratory rates (**c**), and contents of starch and protein (**d**) during the flat-plate culture. Each value is an average ± standard deviation of triplicates (**b**–**d**). **e** Normarski differential interference contrast images of cells at 0, 1, 3, and 6 h in the flat-plate culture. **f** A schematic image of *C. merolae* cell indicating plastid and extra-plastid compartments. Measured transverse and longitudinal diameters of each compartment are also shown. Changes in area (**g**) and aspect ratio (**h**) of plastid and extra-plastid. Each value is an average ± standard deviation of measurement for 30 cells (**g**, **h**)

Previously, we reported a culture method using a flat-plate culture apparatus (Fig. 1a), which alleviates the restriction of light availability caused by the self-shading of cells in a culture of high cell density (Moriyama et al. 2014b). In this study, the physiological characteristics of the cells grown in the apparatus were first determined. Subcultured cells in the stationary phase were concentrated to a culture turbidity (OD_750_) of 10, and then were cultured in a conical flask with rotary shaking overnight to exhaust the intracellular nutrients, such as floridean starch. Then the cells were transferred to the flat-plate culture apparatus and cultured under high light (250 μmol photons m^−2^ s^−1^) with aeration by air containing 1 % CO_2_ at 40 °C for 6 h.

The number of cells remained constant in the culture, although the OD_750_ increased exponentially (Fig. 1b), suggesting that the average cell size or intracellular density increased without cell division in the culture. The thickness of the apparatus was 2 mm, allowing the transmission of 20 and 10 %, respectively, of the incident light from two krypton bulbs (250 μmol photons m^−2^ s^−1^) through the culture apparatus filled with *C*. *merolae* culture at 0 h (OD_750_ = 10) and 6 h (OD_750_ = 18). The turbidity was measured at a distance from the cuvette, whereas the light transmission was measured just behind the plate; therefore, the plate transmittance and the turbidity are not related according to the Lambert–Beer Law. The photosynthetic rate, determined as the rate of oxygen evolution, was slightly increased at 1 h and then gradually decreased to the initial level seen at the beginning of the culture (Fig. 1c). On the other hand, the respiratory rate, determined as the rate of oxygen consumption, increased linearly in proportion to the culture time (Fig. 1c). The ratio of photosynthetic rate to respiratory rate was 29, 7, 4, and 2 at 0, 1, 3, and 6 h, respectively. The starch content of the cells was scarcely detectable at 0 h and then dramatically increased during the culture; thus, the cellular nutritional state changed from poor to enriched during the flat-plate culture. In contrast, the protein content increased only slightly (Fig. 1d), therefore, respiratory and photosynthetic rates were normalized by the protein content in this study.

Figure 1e shows bright field images of the cells in the flat-plate culture. The cells, which began as slender (0 h), became more rounded as the culture proceeded (1–6 h). To further characterize this change in cell shape, each cell was subdivided into a plastid compartment and an extra-plastid compartment containing the cytosol, nucleus, mitochondrion, and other organelles (Fig. 1f), and the areas and the longitudinal/transverse diameters were measured (Fig. 1g, h, Additional file 1: Figure S1). The areas of the plastids remained constant, although the aspect ratios changed. Namely, the plastid shape was longitudinally long in the poor nutrient state (0 h of culture), whereas the shape became transversely long at 1–6 h of culture. In contrast, the area of the extra-plastid space increased during the culture period, while the aspect ratios were constant, with a transversely long shape.

### Subcellular localization of enzymes related to glycerol and lactate metabolism in *C*. *merolae*

**Fig. 2:**
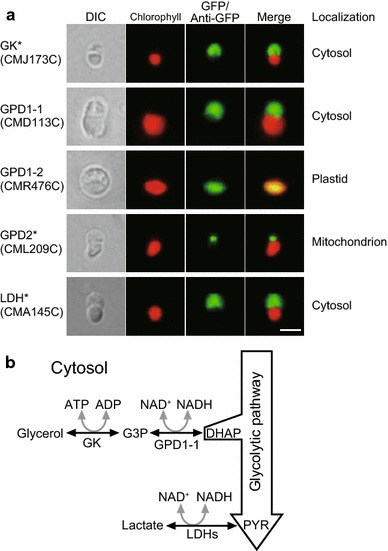
Subcellular localization of GFP-fusion proteins related to glycerol and lactate metabolism in *C. merolae* cells. **a** Fluorescence microscopic images of transiently transformed *C. merolae* cells. *Asterisks* show images observed by immunofluorescence with anti-GFP antibody. *Scale bar* indicates 2 μm. *DIC* Normarski differential interference contrast images, *Chlorophyll* chlorophyll autofluorescence, *Merge* merged images of green fluorescence and chlorophyll autofluorescence. **b** Metabolic pathways for glycerol and lactate in *C. merolae*. *DHAP* dihydroxyacetone phosphate, *G3P* glycerol 3-phosphate, *PYR* pyruvate

Subcellular localization analysis of the enzymes related to carbon metabolism gives information about the cellular metabolic flow within the cell. Previously, we reported on the localization of principal enzymes of the central carbon metabolic pathways (Moriyama et al. 2014a). In this study, we determined the localization of enzymes related to glycerol and lactate metabolism using GFP (Fig. 2a).

Glycerol is reversibly converted into glycerol 3-phosphate (G3P) by glycerol kinase (GK), and then reversibly converted into dihydroxyacetone phosphate (DHAP) by glycerol 3-phosphate dehydrogenase (GPD). Then DHAP is metabolized in the glycolytic pathway. The *C*. *merolae* genome encodes one GK (CMJ173C [protein ID in *Cyanidioschyzon merolae* genome project, http://merolae.biol.s.u-tokyo.ac.jp/]), two NAD^+^-dependent GPDs (GPD1-1, CMD113C; GPD1-2, CMR476C), and one FAD-dependent GPD (GPD2, CML209C). GFP analysis showed cytosolic localization of GK and GPD1-1. GPD1-2 and GPD2, however, were localized to the plastid and mitochondrion, respectively.

Lactate dehydrogenase (LDH) catalyzes the NAD^+^-dependent reversible conversion of pyruvate to lactate. *C*. *merolae* has five LDHs (CMA145C, CMC188C, CMI306C, CMJ002, and CMK006C), which have high sequence similarity with one another. As a representative, we cloned CMA145C into a pCEG vector and found that the LDH was localized to the cytosol. The other LDHs are presumed to also be localized to the cytosol. Together, these results suggest that glycerol and lactate are metabolized, with production of NADH, in the cytosol in *C*. *merolae* (Fig. 2b). The reducing power of cytosolic NADH is presumably transferred to mitochondria via the G3P shuttle, which consists of cytosolic GPD1 and mitochondrial GPD2, as it is in *A*. *thaliana* (Shen et al. 2003).

### Gene expression analysis of central carbon metabolism in *C*. *merolae* cells grown in flat-plate culture

**Fig. 3:**
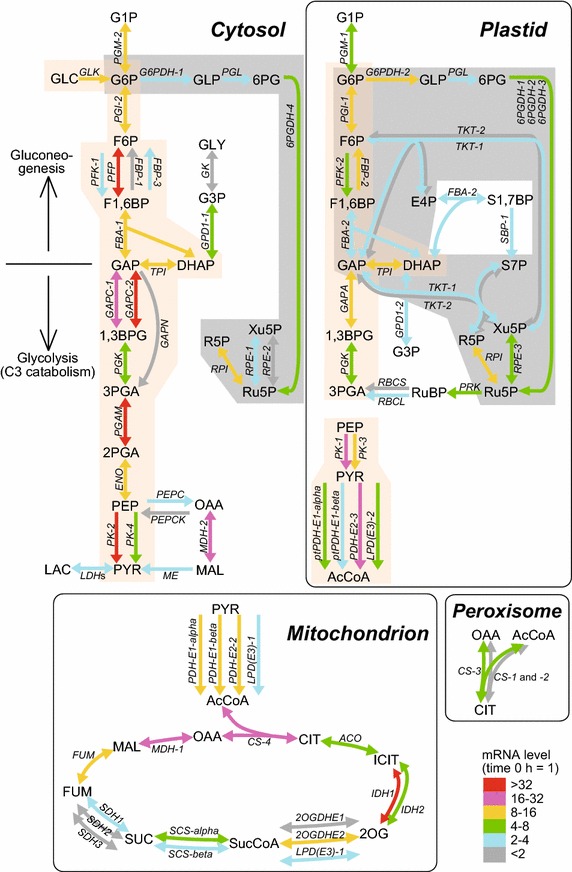
Changes in gene expression in flat-plate culture. Transcript levels of genes related to glycolytic and oxidative pentose phosphate pathways, and Calvin–Benson and citric acid cycles were measured by quantitative RT-PCR. The peak of gene expressions during 0–6 h is *color-coded*. *Explanatory color chart* is shown at the lower right corner of this figure. Transcript level of *SDH4* (succinate dehydrogenase hydrophobic subunit) was not determined because primer pair could not be designed by Primer Express software (Applied Biosystems) for crucially short and AT-rich sequence of *SDH4* gene. Each gene name is shown on the *arrows* corresponding to enzymatic reaction. *Orange* and *gray* backgrounds show glycolytic and oxidative pentose phosphate pathways, respectively. Abbreviations of intermediates: 1,3BPG, 1,3-bisphoglycerate; 2OG, 2-oxoglutarate; 2PGA, 2-phosphoglycerate; 3PGA, 3-phosphoglycerate; 6PG, 6-phosphogluconate; AcCoA, acetyl-CoA; CIT, citrate; DHAP, dihydroxyacetone phosphate; E4P, erythrose 4-phosphate; F1,6BP, fructose 1,6-bisphosphate; F6P, fructose 6-phosphate; FUM, fumarate; G1P, Glucose 1-phosphate; G6P, glucose 6-phosphate; GAP, glyceraldehyde 3-phosphate; GLC, glucose; GLP, glucono 1,5-lactone 6-phosphate; ICIT, isocitrate; MAL, malate; OAA, oxaloacetate; PEP, phosphoenolpyruvate; PYR, pyruvate; R5P, ribose 5-phosphate; Ru5P, ribulose 5-phosphate; RuBP, ribulose 1,5-bisphosphate; S1,7BP, sedoheptulose 1,7-bisphosphate; S7P, sedoheptulose 7-phosphate; SUC, succinate; SucCoA, succinyl-CoA; Xu5P, xylulose 5-phosphate. Function of gene product: *2OGDH*, 2-oxoglutarate dehydrogenase; *6PGDH*, 6-phosphogluconate dehydrogenase; *ACO*, aconitase; *CS*, citrate synthase; *ENO*, enolase; *FBA*, fructose 1,6-bisphosphate aldolase; *FBP*, fructose 1,6-bisphosphatase; *FUM*, fumarase; *G6PDH*, glucose 6-phosphate dehydrogenase; *GAPA*, GAP dehydrogenase (NADP^+^ dependent, phosphorylating); *GAPC*, GAP dehydrogenase (NAD^+^ dependent, phosphorylating); *GAPN*, GAP dehydrogenase (NADP^+^ dependent, non-phosphorylating); *GLK*, glucokinase; *ICDH*, isocitrate dehydrogenase (NADP^+^ dependent); *IDH*, isocitrate dehydrogenase (NAD^+^ dependent); *LPD (E3)*, pyruvate dehydrogenase E3/dihydrolipoamide dehydrogenase; *MDH*, malate dehydrogenase; *ME*, malic enzyme (NADP^+^ dependent); *PDH*, pyruvate dehydrogenase; *PEPC*, phosphoenolpyruvate carboxylase; *PEPCK*, phosphoenolpyruvate carboxykinase; *PFK*, phosphofructokinase (ATP dependent); *PFP*, phosphofructokinase (PPi dependent); *PGAM*, phosphoglycerate mutase; *PGI*, phosphoglucose isomerase; *PGK*, phosphoglycerate kinase; *PGL*, 6-phosphogluconolactonase; *PGM*, phosphoglucomutase; *PK*, pyruvate kinase; *PRK*, phosphoribulokinase; *RBCL*, ribulose bisphosphate carboxylase large subunit; *RBCS*, ribulose bisphosphate carboxylase small subunit; *RPE*, ribulose 5-phosphate 3-epimerase; *RPI*, ribose 5-phosphate isomerase; *SBP*, sedoheptulose 1,7-bisphosphatase; *SCS*, succinyl-CoA synthetase; *SDH1*, succinate dehydrogenase (Complex II) flavoprotein subunit; *SDH2*, succinate dehydrogenase iron-sulfur protein; *SDH3*, succinate dehydrogenase cytochrome B560 subunit; *TKT*, transketolase; *TPI*, triosephosphate isomerase

The transcript levels of genes related to central carbon metabolism, including the glycolytic and oxidative pentose phosphate pathways, as well as the Calvin–Benson and citric acid cycles, were measured using quantitative RT-PCR at 0, 1, 3, and 6 h in the flat-plate culture (Additional file 1: Figure S2). In the assay, 12 genes showed constant transcript levels (Additional file 1: Figure S2a). The transcript levels of the other 70 genes increased 2- to 110-fold at 1 h and then remained constant to 6 h (Additional file 1: Figure S2b–f). Figure 3 shows a result of expression mapping of the carbon metabolic pathways, based on their subcellular localizations determined previously (Moriyama et al. 2014a) and in this study (Fig. 2). The extent of the increases in gene expression, compared to the level at 0 h, is color-coded. Four of the enzymes [6-phosphogluconolactonase (PGL), triosephosphate isomerase (TPI), phosphoglycerate kinase (PGK), and ribose-5-phosphate isomerase (RPI)] are localized to both the cytosol and plastid.

The transcript levels of many of the cytosolic glycolytic genes increased dramatically during the culture. In particular, the transcript levels of the genes for PPi-dependent phosphofructokinase (PFP), glyceraldehyde 3-phosphate dehydrogenase (GAPC-2), phosphoglycerate mutase (PGAM), and pyruvate kinase (PK-2), increased 37-, 45-, 62-, and 35-fold, respectively (Additional file 1: Figure S2f). The increase in expression of the plastid glycolytic genes was relatively less than that of the cytosolic glycolytic genes. In the plastid glycolytic genes, the transcript levels of *PK*-*1* and *PDH*-*E2*-*3*, the protein products of which are related to synthesis of acetyl-CoA used in fatty acid synthesis, increased dramatically. The complete oxidative pentose phosphate pathway is localized to the plastid, but a partial pathway is also present in the cytosol. The variation in transcript levels of these genes was small. The transcript levels of the Calvin–Benson cycle genes, some of which are shared by the oxidative pentose phosphate pathway, increased relatively little. Among the citric acid cycle genes, the transcript levels of *MDH*-*1*, *CS*-*4*, and *IDH1* increased 22-, 20-, and 110-fold, respectively. The *C*. *merolae* genome encodes three peroxisomal citrate synthase genes, *CS*-*1*, *CS*-*2*, and *CS*-*3*, which are believed to be involved in the metabolism of acetyl-CoA produced by β-oxidation of fatty acids (Moriyama et al. 2014a); increases in the transcript levels of these peroxisomal *CS* genes were less than that of mitochondrial *CS*-*4*. These results suggest that the regulation of expression of at least some of the cytosolic glycolytic and citric acid cycle genes, which were severely suppressed in the poor nutrient state (0 h of culture), was related to the regulation of the respiratory rate, whereas the genes of photosynthetic proteins remained at a constant level of expression in *C*. *merolae* cells, regardless of the amount of photosynthetic products, to maintain a constant rate of photosynthesis.

### Effects of exogenous organic compounds on the respiratory rate in nutrient-deficient cells

**Fig. 4:**
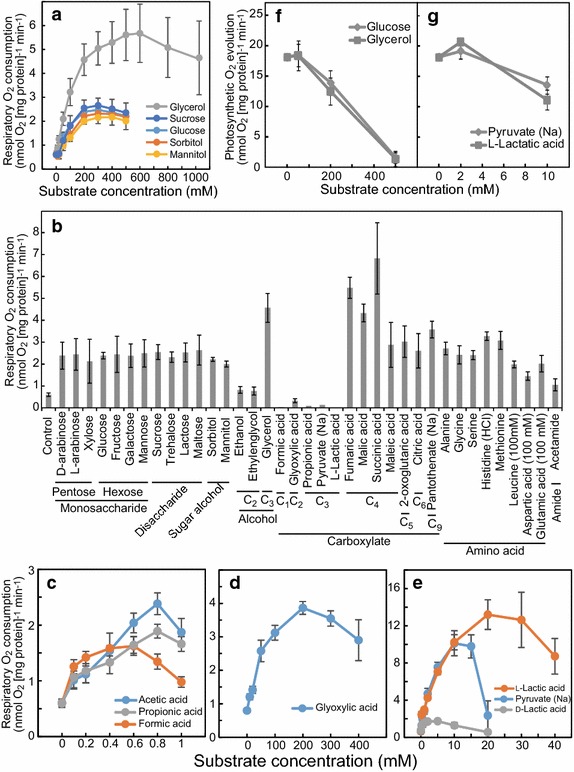
Effects of exogenous organic substances on respiratory and photosynthetic rates in nutrient-deficient cells. Organic substrates were added to culture at 0 h in the flat-plate culture. **a** Respiratory oxygen consumption rate (respiratory rate) dependent on sugar concentration. **b** Respiratory rate with addition of organic substances at 200 mM. Leucine, aspartic acid, and glutamic acid were added to the culture at 100 mM for their low aqueous solubility. Respiratory rate dependent on concentration of acetic acid, propionic acid, and formic acid (**c**), glyoxylic acid (**d**), and l-lactic acid, pyruvate, and d-lactic acid (**e**). Photosynthetic oxygen evolution rate dependent on concentration of glucose and glycerol (**f**) and pyruvate and l-lactic acid (**g**). Each value is an average ± standard deviation of triplicates

To evaluate the physiological effects of exogenous organic compounds in *C*. *merolae* cells, we measured the respiratory rate in cells grown in a shaken culture with high cell density (OD_750_ = 10), corresponding to t = 0 in the flat-plate culture. Then, changes in the respiratory rate as a function of substrate concentration were evaluated for glycerol, sucrose, glucose, sorbitol, and mannitol (Fig. 4a). Sucrose might be partially hydrolyzed to glucose and fructose in 2 × Allen’s medium at low pH (pH 2.5). Each of the added substrates increased the respiratory rate, with glycerol having the greatest effect. The respiratory rate plateaued at 200 mM sugar for sucrose, glucose, sorbitol, and mannitol, and at 600 mM glycerol, and then decreased with higher concentrations.

Based on these results, various substrates, including sugars, alcohols, carboxylic acids, and amino acids, were added to the cells at 0 h in the flat-plate culture at a concentration of 200 mM (Fig. 4b). Due to the low solubility of leucine, aspartate, and glutamate, these amino acids were added at a concentration of 100 mM. Stock solutions of fumaric acid, succinic acid, and pantothenate were adjusted to pH 5.2, pH 4.5, and pH 5.0, respectively, and solutions of the other carboxylic acids and amino acids were adjusted to pH 2.5. Monosaccharides, disaccharides, sugar alcohols, carboxylic acids (maleic acid, 2-oxoglutaric acid, citric acid, and pantothenate), and amino acids increased the respiratory rate 3- to 6-fold, whereas ethanol, ethylene glycol, and acetamide increased the respiratory rate 2-fold. Glycerol and four-carbon-atom carboxylic acids (fumaric acid, malic acid, and succinic acid) increased the respiratory rate 7- to 11-fold. Carboxylic acids with a carbon number less than three proved toxic to *C*. *merolae* cells, which immediately turned yellow and died when the concentration of carboxylic acids was 200 mM.

To better characterize the effects of short-chain carboxylic acids on the respiratory rate, these substrates were added to the culture at low concentration (Fig. 4c–e). Upon addition of 1 mM acetic acid, propionic acid, or formic acid, *C*. *merolae* cells survived, and the respiratory rate increased 3- to 4-fold (Fig. 4c). Glyoxylic acid could be added to the culture up to 400 mM, and the respiratory rate increased 4-fold at a concentration of 200 mM. Figure 4b, d show the contrasting effects of 200 mM glyoxylic acid on the respiratory rate, probably due to differences in the method of substrate addition. In the assay of Fig. 4b, glyoxylic acid was added to the culture to a final concentration of 200 mM at once, whereas in Fig. 4d the glyoxylic acid was added step-wise to final concentrations of 10, 20, 50, 100, 200, 300, and 400 mM, with 10 min intervals between additions. Thus, the cells appear to have acclimated to the glyoxylic acid in Fig. 4d. l-Lactic acid and pyruvate increased the respiratory rate 19- and 16-fold, respectively (Fig. 4e). d–Lactic acid had virtually no effect on the respiratory rate (Fig. 4e), suggesting that l-lactic acid was specifically metabolized by lactate dehydrogenases in *C*. *merolae*.

Assuming that respiratory oxygen consumption behaves as a single enzyme-catalyzed reaction, we applied Michaelis–Menten kinetics to the substrate concentration-dependent respiratory rates in the results shown in Fig. 4a, c–e (Table 1). Because cells were assumed to contain internal substrates for respiration, a parameter was added to account for the concentration of intracellular substrate (*S*_in_); thus, the modified Michaelis–Menten equation was $v = {V_{ \text{max} }} \cdot \left( {\left[ S \right] + \left[ {S_{\text{in}} } \right]} \right)/K_{\text{s}} + \left( {\left[ S \right] + \left[ {S_{\text{in}} } \right]} \right)$. *K*_s_ indicates the apparent *K*_m_; that is, the substrate concentration (*S*) at which the rate of respiration was half maximal (i.e., *v* = 1/2*V*_max_). *V*_max_ values were high for l-lactic acid, pyruvate, and glycerol. The *K*_s_ values for carboxylic acids with a carbon number less than three were smaller than those for sugars and glycerol. Taken together, we interpret these results as follows: sugars, which are catabolized in the early steps of glycolysis, increased the respiratory rate approximately 4-fold; glycerol, which is catabolized in a middle step of glycolysis, increased the rate 9-fold; pyruvate and l-lactic acid, both end products of glycolysis, increased the respiratory rate 16- or 19-fold; and fumaric acid, malic acid, and succinic acid, which are metabolized in the citric acid cycle, increased the respiratory rate 7- to 11-fold. These observations suggest that the further downstream the organic substance is catabolized in the carbon metabolic pathway, the greater its effect on the respiratory rate.

**Table 1:** Kinetics for exogenous substrates on respiratory rate in nutrient-deficient cells

Substrates	*V*_max_	*K*_s_ (mM)	*S*_in_ (mM)	*R* ^2^
l-Lactic acid	16.3 ± 1.2	6.7 ± 1.7	0.6 ± 0.3	0.94
Pyruvate (Na)	12.8 ± 0.9	3.7 ± 0.8	0.1 ± 0.1	0.96
Glycerol	7.1 ± 0.6	132 ± 34	10 ± 5	0.94
Glyoxylic acid	4.4 ± 0.2	47 ± 10	9 ± 3	0.95
Sucrose	4.3 ± 0.8	174 ± 82	21 ± 10	0.91
Mannitol	4.2 ± 1.1	296 ± 164	41 ± 16	0.93
Sorbitol	4.1 ± 0.4	231 ± 51	29 ± 5	0.97
Glucose	3.4 ± 0.2	105 ± 20	17 ± 4	0.96
Acetic acid	3.0 ± 0.6	0.43 ± 0.25	0.10 ± 0.06	0.84
Propionic acid	2.2 ± 0.2	0.24 ± 0.12	0.01 ± 0.05	0.84
Formic acid	1.8 ± 0.1	0.05 ± 0.03	0.03 ± 0.01	0.88
d-Lactic acid	1.8 ± 0.1	0.13 ± 0.07	0.1 ± 0.1	0.76

Kinetic parameters were calculated by fitting to the modified Michaelis–Menten equation: $v = V_{\text{max} } \left( {\left[ S \right] + \left[ {S_{\text{in}} } \right]} \right)/K_{\text{s}} + \left( {\left[ S \right] + \left[ {S_{\text{in}} } \right]} \right)$. *V*_max_ (nmol O_2_ [mg protein]^−1^ min^−1^) indicates maximal velocity of respiration. *K*_s_ indicates the substrate concentration which corresponds to the half of *V*_max_. *S* means the concentration of exogenous substrate. *S*_in_ means hypothetical concentration of intracellular substrate. *R* ^2^ indicates coefficient of determination. Substrates are shown in descending order for the value of *V*_max_. Each value is an average ± standard deviation of triplicates

Next, we examined the effects of glucose, glycerol, pyruvate, and l-lactic acid on photosynthetic oxygen evolution (Fig. 4f, g). The photosynthetic rate was not affected by 50 mM glucose or glycerol (Fig. 4f), but decreased when these substrates were present at a concentration greater than 200 mM. The photosynthetic rate increased slightly in the presence of 2 mM pyruvate or l-lactic acid, but decreased when these carboxylic acids were present at a concentration of 10 mM (Fig. 4g). These results indicate that these organic substances have different effects on photosynthesis than on respiration.

### Effects of exogenous organic substances on respiration in *C*. *merolae* cells at different flat-plate culture times

**Fig. 5:**
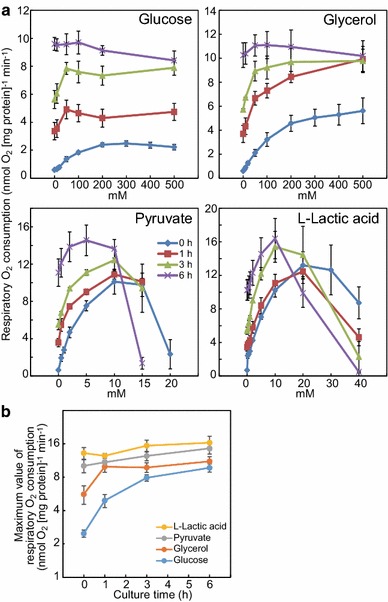
Effects of organic substances on respiratory rate in *C. merolae* cells at different flat-plate culture times. **a** Glucose, glycerol, pyruvate, and l-lactic acid were added to culture at 0 (*blue*), 1 (*red*), 3 (*light green*), and 6 h (*purple*) in the flat-plate culture, and respiratory rate was measured. Results at 0 h are adopted from the results of Fig. 4 in each substance. Each value is an average ± standard deviation of triplicates. **b** From the results of (**a**), maximum values of respiratory rates at different culture times are plotted. *Vertical scale* is indicated as logarithmic scale

To examine the effects of exogenous compounds on respiration in *C*. *merolae* cells with different levels of starch accumulation, glucose, glycerol, pyruvate, and l-lactic acid were added to cells grown in the flat-plate culture at 0, 1, 3, and 6 h, and the respiratory rates were measured (Fig. 5a). In the kinetics assays (Table 1), we attempted, but were unable, to fit the large respiratory rates at 1, 3, and 6 h of flat-plate culture in the absence of added substrates to the modified Michaelis–Menten equation.

In the presence of glucose, the respiratory rate of nutrient-deficient cells (culture time of 0 h) continued to increase up to a glucose concentration of 200 mM, whereas in cells cultured for 1 or 3 h, the respiratory rates plateaued at a glucose concentration of 50 mM. Similar results were observed for glycerol. In cells cultured for 6 h, the respiratory rate was unaltered by even high concentrations of glucose, whereas a slight increase was observed upon addition of exogenous glycerol. In addition, the maximum respiratory rate in the presence of glucose varied, depending on the culture time. In contrast with the effects of glucose and glycerol, the effects of pyruvate and l-lactic acid were similar among the cells at 0, 1, 3, and 6 h of culture, but the optimum concentrations of these carboxylic acids were lower at later time points, at which the respiratory rate was higher. In addition, pyruvate and l-lactic acid increased the respiratory rate in cells cultured for 6 h, and the maximum respiratory rates were similar at all culture times. Judging from the differences observed between the effects of the four exogenous substrates on the respiratory rates at 6 h of culture, there seem to be at least two rate-limiting steps in glycolysis, as measured by respiratory activity; namely, one between glucose 6-phosphate (G6P) to glyceraldehyde 3-phosphate (GAP), and one between GAP to pyruvate.

Figure 5b shows plots of the maximum respiratory rate upon addition of four substances at different times of flat-plate culture. The plots can be interpreted as reflecting changes in the maximum activities of various portions of the carbon metabolic pathways. For example, the assay with exogenous glucose presumably measures the activity of the glycolytic pathway from G6P to GAP, while the assay with exogenous glycerol measures the activity from GAP to pyruvate, and the assay with exogenous pyruvate and l-lactic acid measures the activity of the downstream citric acid cycle. Interpreted from this viewpoint, the activity of the first half of glycolysis (G6P to GAP) increased dramatically during the flat-plate culture, while the activity of the latter half of glycolysis (GAP to pyruvate) reached a maximum at 1 h of flat-plate culture. In contrast, the increase in activity of the downstream citric acid cycle was relatively small.

### Effects of exogenous substrates on growth

To examine whether the increase in respiratory rate induced by exogenous substrates was correlated with an increased rate of cell growth, glycerol, succinic acid (pH 4.5), and l-lactic acid (pH 2.5) were added to the culture to final concentrations of 50, 50, and 5 mM, respectively, and cell growth rates were determined (Table 2). At these concentrations, these substrates effectively increased the respiratory rate but did not interfere with photosynthetic activity (Fig. 4f, g). The growth rates were measured under two conditions, one at low light intensity (8 μmol photons m^−2^ s^−1^) while bubbling with ordinary air, and another at intermediate light intensity (50 μmol photons m^−2^ s^−1^) while bubbling with air containing 1 % CO_2_. Under the former condition, relative to the control (without a carbon source), the growth rate increased and the doubling time was shortened by 4.6, 7.4, and 10.2 h with exogenous glycerol, succinic acid, and l-lactic acid, respectively. The order of effectiveness of the substrates on growth rate paralleled their effectiveness at increasing the respiratory rate; thus, l-lactic acid showed the highest effect on growth rate among the substrates examined under conditions of insufficient photosynthesis. The doubling time of the control was shorter under the second condition than under the first condition, and the growth rate was barely affected by the addition of exogenous substrates.

**Table 2:** Mixotrophic growth rate in *C. merolae*

Added carbon source	Doubling time (h)	
	Light: 8 μmol photons m^−2^ s^−1^ Aeration: air	Light: 50 μmol photons m^−2^ s^−1^ Aeration: 1 % CO_2_
None	35.6 (±2.1)	12.7 (±0.5)
50 mM glycerol	31.0 (±3.1)	11.8 (±0.4)
50 mM succinic acid	28.2 (±2.7)*	12.2 (±0.3)
5 mM l-lactic acid	25.2 (±2.5)*	12.0 (±0.2)

Each value is an average ± standard deviation of triplicates

* *p* < 0.05 in *t* test (compared with control)

### Heterotrophic growth in *C*. *merolae*

**Fig. 6:**
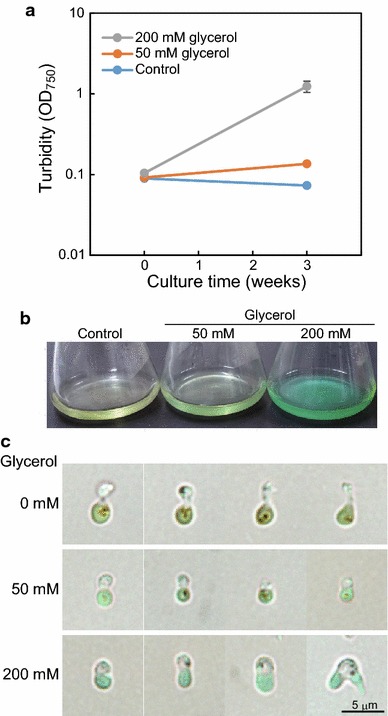
Heterotrophic growth in *C. merolae*. *C. merolae* cells were grown with addition of 0, 50, and 200 mM glycerol in rotary-shaken flasks under the darkness for 3 weeks. **a** Turbidity of dark-grown culture. Culture images in flasks (**b**) and microscopic images (**c**) after 3-week. In **c**, four cells are shown as representative. A dividing cell is shown on the extreme right at 200 mM glycerol

*Cyanidioschyzon merolae* was supplied with 200 mM glucose, 200 mM glycerol, 200 mM succinic acid, or 5 mM lactic acid and cultured in flasks shaken in the dark (Additional file 1: Figure S3). The *C*. *merolae* cells started to grow exponentially after 1 week in the presence of glycerol, although the doubling time was very long, approximately 1 week. In the presence of other substrates or no substrate (control), the turbidity of the culture continued to decrease over 4 weeks. In this assay, the cell cultures were exposed to room light at the sampling time for a short period of time. Next, the cells were cultured under complete darkness in a medium containing 50 or 200 mM glycerol, and the turbidity was measured 3 weeks later, demonstrating that *C*. *merolae* could grow heterotrophically with 200 mM glycerol (Fig. 6a, b). Under microscopic observation, the dark-grown cells in the presence of glycerol appeared to be normal in shape (Fig. 6c). On the other hand, the dark-grown cells without exogenous glycerol exhibited an abnormal shape with an extremely contracted cytosol and/or contraction between the cytosol and plastid (Fig. 6c).

## Discussion

### Culture of *C*. *merolae* in the flat-plate culture apparatus

We used the flat-plate culture system to measure respiratory rate in *C*. *merolae* cells with different levels of starch accumulation at high cell densities because effects of exogenous substrates on respiratory rate were not measurable if the cells were in the logarithmic phase (OD_750_ <1). To obtain cells with a wide range of starch accumulation levels, the flat-plate culture was started using a shaken culture (OD_750_ = 10). The cells at 0 h contained hardly any starch and did not undergo division, suggesting that the cell cycle was quiescent (i.e., in the G0 phase), and the cells reentered the G1 phase during the flat-plate culture with high-light irradiation. In the green alga *Chlamydomonas reinhardtii*, the protein compromised hydrolysis of triacylglycerols 7 (CHT7), which contains a DNA binding domain and is localized to the nucleus, is related to quiescence with respect to cellular nutritional status (Tsai et al. 2014). In synchronous culture of *C*. *merolae*, cell division occurred at 10–12 h from the beginning of the light phase (Fujiwara et al. 2009; Moriyama et al. 2010), and therefore cells in the flat-plate culture will divide if the culture time is prolonged.

When physiological changes were measured in the flat-plate culture, the variance was small, indicating that individual cells were evenly irradiated by light. The flat-plate culture system consists of materials used commonly for gel electrophoresis, along with thin silicon tubes for aeration; thus, no special materials are required, and the culture system can be easily acquired by any laboratory. In addition, the system can be applied to other algae, as well as *C*. *merolae*.

### Utilization of exogenous organic substances by *C*. *merolae*

As far as we examined, *C*. *merolae* could utilize almost any exogenous organic substance as a respiratory substrate (Fig. 4). In contrast, exogenous glucose and glycerol did not increase the photosynthetic rate, and excess substrate actually decreased this rate. The decrease in photosynthetic rate by addition of glucose and glycerol at high concentrations may have been caused by a rapid change in the osmotic pressure. Pyruvate and lactic acid slightly stimulated photosynthetic activity at low concentrations. Because photosynthetic rate is related to respiratory rate (Vedel et al. 1999), it is possible that in our study increased respiratory activity stimulated the photosynthetic activity. These results indicate that exogenous substrates were indeed imported into the cells. *C*. *merolae* encodes a single gene of sugar transporter (CMK066C). In *A. thaliana*, the monosaccharide/proton symporter AtSTPs, which have sequence similarity with CMK066C, transport various sugars, including glucose, fructose, galactose, mannose, xylose, and arabinose, into the cell (Truernit et al. 1999; Sherson et al. 2000). In *C*. *merolae*, monosaccharides were presumably taken up by the function of the sugar transporter, although the efficiency of uptake of exogenous sugars was low (i.e., the *K*_s_ values for sugars were large, Table 1). Exogenous glycerol dramatically increased the respiratory rate, although the *C*. *merolae* genome encodes no glycerol permease, unlike *G*. *sulphuraria* (Barbier et al. 2005). The cell membrane is highly permeable to glycerol, and glycerol permease may not be required for glycerol uptake in *C*. *merolae*, which lacks a cell wall (Oesterhelt et al. 2008). Short-chain carboxylic acids with a carbon number less than three are not significantly ionized in 2 × Allen’s medium because the acid dissociation constant (p*K*_a_) of these carboxylic acids is much greater than the pH value of the medium (pH 2.5). Therefore, the short-chain carboxylic acids should easily diffuse into *C*. *merolae* cells, and indeed, the *K*_s_ values for these acids were much lower than those for the sugars (Table 1). Because of this high permeability, when short-chain carboxylic acids were added to the culture at a high concentration, it is plausible that the carboxylic acids rapidly entered the cells, and then, encountering a neutral pH (Zenvirth et al. 1985), ionized to release excess protons, resulting in cell death.

Under mixotrophic conditions, the efficiency with which exogenous substrates affected the growth rate in *C*. *merolae* was different than in the algae *G*. *sulphuraria* (Oesterhelt et al. 2007) and *Chlamydomonas acidophila* (Tittel et al. 2005), which can grow heterotrophically. In these algae, the growth rate effectively increased upon addition of carbon sources, even when the algae were irradiated with sufficient light intensity for photosynthesis. These results suggest that *C*. *merolae* cells with high photosynthetic activity can undergo cell division. We also demonstrated heterotrophic growth in *C*. *merolae* in the presence of glycerol (Fig. 6, Additional file 1: S3). Exogenous glycerol was presumably converted into DHAP or GAP in the cytosol. Because GAP is a product of photosynthesis and is transferred into the cytosol from the plastid under lighted conditions, a large amount of GAP derived from exogenous glycerol might mimic the state of cells actively performing photosynthesis. Accordingly, genes necessary for progression of the cell cycle might be transcribed in cells supplied with exogenous glycerol in darkness. The failure to observe heterotrophic growth with exogenous glucose, l-lactic acid, and succinic acid might also be explained by the level of GAP. Exogenous glucose would hardly be catabolized into GAP if the activities of enzymes in the first half of glycolysis, such as glucokinase, PFK, or fructose 1,6-bisphosphate aldolase, are suppressed in darkness. For l-lactic acid and succinic acid, these substrates are catabolized in the citric acid cycle, and metabolism of these substrates is not related to that of GAP. For verification of this hypothesis, transcriptome and metabolome analyses are needed.

Our results demonstrate that *C*. *merolae* can grow in darkness with 200 mM glycerol. Similar results have been reported for the haptophyte *Prymnesium parvum* (Rahat and Jahn 1965) and marine cryptomonad *Chroomonas salina* (Antia et al. 1973); these algae can grow heterotrophically with 250 mM exogenous glycerol. However, ecologically speaking, these algae are considered to be obligate photoautotrophs because very high concentrations of glycerol do not normally exist in their natural habitat (Droop 1974). Similarly, *C*. *merolae* may be considered an ecologically obligate photoautotroph.

### Regulation of respiration in *C*. *merolae*

**Fig. 7:**
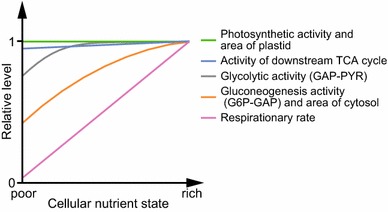
Diagram showing the regulation of the activity of respiration and photosynthesis in *C. merolae*. Details of this diagram are explained in the last part in “Discussion” section

Figure 7 summarizes the results of this study. During flat-plate culture, the respiratory rate increased dramatically, while the photosynthetic rate was constant, irrespective of starch accumulation levels. The results of Fig. 5 suggest that glycolysis was rate-limiting to respiration. The respiratory process was subdivided into three pathways, namely, the pathway from G6P to GAP in glycolysis (G6P-GAP pathway), the pathway from GAP to pyruvate in glycolysis (GAP-PYR pathway), and the downstream pathway of the citric acid cycle. The GAP-PYR pathway was immediately activated in response to enrichment of the cellular nutritional status, which may reflect the necessity to rapidly catabolize GAP, which is a photosynthetic product and should be consumed as a respiratory substrate to maintain photosynthesis. The G6P-GAP pathway also increased, but the increase was milder than that in the GAP-PYR pathway and the increase continued throughout the 6 h of culture. The G6P-GAP pathway is used for both catabolism and anabolism of starch, which functions as storage for excess energy. The production of ATP and metabolites responsible for biosynthesis through the downstream GAP-PYR pathway might be prioritized over starch metabolism if the nutrient status of the cells is very low. The results of the gene expression analysis suggest that the regulation of the *PFP* transcript level is related to the regulation of the G6P-GAP pathway, and the regulation of the transcript levels of *GAPC*, *PGAM*, and *PK* are related to the regulation of the GAP-PYR pathway. In plants, the expression level of PFP (Groenewald and Botha 2007) and GAPC (Zaffagnini et al. 2013) is related to the regulation of the respiratory rate. In contrast to glycolytic activity, the activity of the downstream citric acid cycle was largely unaltered, although some citric acid cycle genes showed large changes in expression level during the flat-plate culture. Upon increase in the activity of these metabolic pathways, the respiratory rate increased dramatically during the flat-plate culture, indicating that the amount of respiratory substrate limits the respiratory rate and has an influence on the ratio of photosynthetic rate to respiratory rate.

We found that cell size and shape also changed during the flat-plate culture. Changes in the areas of the plastid and extra-plastid compartments reflect the photosynthetic and glycolytic (G6P-PYR) activities, respectively. Because starch is synthesized and accumulated in the cytosol in red algae, the increase in the area of the extra-plastid compartment appears to be due mainly to an increase in the accumulation of starch. Alternatively, the development of cytosolic glycolysis and/or mitochondrial TCA cycle/electron transfer system may contribute to the increase in the area of the extra-plastid space. Because *C*. *merolae* has no cell wall, the shape and size of the cell may be variable in response to cellular nutritional states.

## Conclusions

*Cyanidioschyzon merolae* utilizes exogenous organic substances as substrates for respiration, the rate of which is mainly regulated by modulation of cytosolic glycolytic activity in response to the level of starch accumulation. This activity seems to metabolize the photosynthetic product GAP to maintain the rate of photosynthesis. Exogenous organic compounds indeed accelerate the growth of *C*. *merolae* under mixotrophic conditions. Furthermore, we demonstrated that *C*. *merolae*, which was thought to be an obligate photoautotroph, can heterotrophically grow in medium containing glycerol, but not other organic substances. The cellular state in which GAP is synthesized from exogenous glycerol may mimic the state of cells actively performing photosynthesis.

## Methods

### Culture conditions of *C. merolae*

*Cyanidioschyzon merolae* strain 10D (Toda et al. 1998) was grown in 100 mL 2 × Allen’s medium (pH 2.5; Minoda et al. 2004) in 200-mL flasks. Flasks were shaken under continuous illumination provided by fluorescent tubes at a fluence rate of 40 µmol photons m^−2^ s^−1^ at 40 °C. For measurement of growth rate, *C. merolae* cells, to which were added 50 mM glycerol, 50 mM succinic acid (pH 4.7), or 5 mM lactic acid (pH 2.5), were grown under continuous light provided by LED lighting system at a fluence rate of 8 µmol photons m^−2^ s^−1^ with aeration by ordinary air or under continuous light at a fluence rate of 50 µmol photons m^−2^ s^−1^ with aeration by air containing 1 % CO_2_ at 40 °C. Because cells died if 5 mM lactic acid was added to a culture at a time, lactic acid was added stepwise; namely, 0.2, 0.6, 1, 2, and 5 mM lactic acid (final concentrations) were added to the culture at every 2 min. OD_750_ of cultures was measured over 2 days, and doubling time was calculated.

### Culture of *C. merolae* in a flat-plate culture apparatus

Subcultured cells (200 mL) were grown to OD_750_ = 4–5 with aeration by 1 % CO_2_ at 40 °C, and centrifuged at 4000*g* for 5 min at 35 °C. The precipitated cells were resuspended in 50 mL 2 × Allen’s medium to OD_750_ = 10 in a 100-mL flask. The concentrated culture in the flask was shaken under continuous light at a fluence rate of 40 µmol photons m^−2^ s^−1^ at 40 °C overnight. The culture was transferred into a flat-plate culture apparatus (Moriyama et al. 2014b), which was assembled from materials for gel electrophoresis consisting of two glass plates with a silicone spacer (clearance of 2 mm), equipped with two silicone tubes (0.8 mm wall, Bio-Rad Laboratories, Hercules, CA, USA) for aeration. The cells were cultured in the flat-plate culture apparatus under high light provided by two 20 W krypton bulbs (250 µmol photons m^−2^ s^−1^) with aeration by 1 % CO_2_ at 40 °C.

### Construction of *GFP*-fusion gene

For the construction of *N*-terminal fusion of EGFP with metabolic enzymes expressed by the *APCC* promoter, first, the *EGFP* gene fragment, which was amplified by PCR with pEGFP vector (Clontech Laboratories, Mountain View, CA, USA) as a template with primers [5′-ACCTCTAGAGGATCCATGGTGAGCAAGGGCGAGGA-3′ and 5′-AGCCGGGCGGCCGCTTTACTTGTACAGCTCGTCCA-3′; underlined sequences are required for cloning using the In-Fusion reaction (Clontech Laboratories)], was inserted into DNA fragment containing the *APCC* promoter and *NOS* terminator, which was amplified by PCR with pCG1 vector (Watanabe et al. 2011) as a template with primers (5′-GGATCCTCTAGAGGTCAACGAACGAAGAAACACAG-3′ and 5′-AGCGGCCGCCCGGCTGCAGATCGTTCAAACATTTG-3′) using In-Fusion HD cloning kit (Clontech Laboratories), and the plasmid was named pCEG1 vector. Metabolic genes were amplified by PCR with specific primer sets; for NAD^+^-dependent glycerol 3-phosphate dehydrogenase (GPD1-1, CMD113C), 5′-TCGTTGACCTCTAGAATGACGGAGAAACATAAGGT-3′ and 5′-CATGGATCCTCTAGACGATTCTGTCTTCGTTTTGA-3′; for GPD1-2 (CMR476C), 5′-TCGTTGACCTCTAGAATGCAGCCTGAGCAAACTGT-3′ and 5′-CATGGATCCTCTAGAACCATTGTTAGCCAAGGCAA-3′; for FAD-dependent glycerol 3-phosphate dehydrogenase (GPD2, CML209C), 5′-TCGTTGACCTCTAGAATGATGCCCTGCGTTCGTAT-3′ and 5′-CATGGATCCTCTAGAGTCGAGTGCAACGCCGGAGC-3′; for glycerol kinase (CMJ173C), 5′-TCGTTGACCTCTAGAATGAAAGAGAAACGCTTTGC-3′ and 5′-CATGGATCCTCTAGAAATGTCGGCAGGCTTTATGC-3′; for lactate dehydrogenase, 5′-TCGTTGACCTCTAGAATGACGTCGGGGATCGACGA-3′ and 5′-CATGGATCCTCTAGATCGGGTCAAAATATTATAGG-3′. Underlined sequences in primer sequences are required for cloning using the In-Fusion reaction. The amplified DNA fragments were inserted into *Xba*I-cut pCEG1 vector with In-Fusion HD cloning kit. *C. merolae* encodes five lactate dehydrogenases, which have high sequence similarity with each other, and the cloned lactate dehydrogenase was determined as CMA145C of protein ID by DNA sequencing.

### Transformation of *C. merolae* cells and observation of subcellular localization

*Cyanidioschyzon merolae* cells were transformed by the PEG-method according to Moriyama et al. (2014b). Observation of EGFP-fusion proteins with immunofluorescence detection using anti-GFP antibody was performed as previously reported (Moriyama et al. 2014a).

### RNA purification and quantitative RT-PCR

Preparation of total RNA and quantitative RT-PCR were performed as described in Moriyama et al. (2010). Briefly, total RNA was prepared by RNeasy Plant Mini kit and RNase-Free DNase Set (Qiagen, Hilden, Germany). First-strand cDNA was prepared using 0.5 μg of total RNA and random primers by SuperScript III reverse transcriptase (Invitrogen, Carlsbad, CA, USA) and Recombinant Ribonuclease Inhibitor (Takara, Otsu, Japan). Quantitative PCR was performed using Power SYBR Green PCR Master Mix (Applied Biosystems, Foster City, CA, USA) and appropriate primers in a Real-time PCR system (model 7300, Applied Biosystems). The primers, which were designed by Primer Express software (Applied Biosystems), are listed in Additional file 1: Table S1. The 18S rRNA gene was used to normalize transcript abundance.

### Measurement of respiration and photosynthesis

Rates of respiration and photosynthesis of *C. merolae* culture were measured polarographically at 40 °C in Oxytherm with an Oxygraph controller (Hansatech Instruments Ltd., Norfolk, UK). Cells were irradiated by high light (6000 µmol photons m^−2^ s^−1^) during the measurement of photosynthetic rate. For kinetic analysis, curve fitting was performed with the modified Michaelis–Menten equation: $v = V_{ \text{max} } \cdot\left( {\left[ S \right] + \left[ {S_{\text{in}} } \right]} \right)/K_{\text{s}} + \left( {\left[ S \right] + \left[ {S_{\text{in}} } \right]} \right)$ by the software SigmaPlot 12.5 (Systat Software, San Jose, CA, USA). *V*_max_ (nmol O_2_ [mg protein]^−1^ min^−1^) indicates maximal velocity of respiration. *K*_s_ indicates the substrate concentration which corresponds to the half of *V*_max_. *S* means the concentration of exogenous substrate. *S*_in_ means hypothetical concentration of intracellular substrate.

### Measurement of floridean starch and protein contents

Quantitation of starch was performed using Glycogen Colorimetric Assay kit II (BioVision, Milpitas, CA, USA). For sample preparation, 50 μL of *C. merolae* culture grown in a flat-plate culture apparatus was collected by centrifugation at 1200*g* for 5 min, and the precipitated cells were resuspended in 90 % ethanol (v/v), and centrifuged at 10,000*g* for 2 min. This wash process was repeated twice. The washed cells containing starch were completely lysed with 100 μL of 10 N KOH at 100 °C for 5 min and were neutralized with 26 μL of 43.8 N H_3_PO_4_. The solubilized starch solution was diluted into 20-fold with the Glycogen Hydrolysis Buffer in the (Glycogen Colorimetric Assay) kit. Subsequent manipulation was performed according to the protocol of the Glycogen Colorimetric Assay kit II. Protein content was measured by the Lowry method.

### Measurement of cell geometry

*Cyanidioschyzon merolae* cells were observed without fixation under a fluorescence microscope BX-60 (Olympus, Tokyo, Japan). Nomarski differential interference image was recorded by a digital camera (model DP-70, Olympus) and the area and the length of plastid and extra-plastid compartments were measured by ImageJ software (http://rsb.info.nih.gov/ij/).

## Additional file

**Additional file 1:** **Table S1**. List of primers for quantitative RT-PCR. All primers were designed by Primer Express software (Applied Biosystems). Slope and amplification efficiency (E) of each primer pair in quantitative-PCR are also shown. **Figure S1** Changes in cell size and shape of *C. merolae* cell in the flat-plate culture. (a) A plot for areas of a plastid and an extraplastid at 0, 1, 3, and 6 h in the culture. Compartmentation of the plastid and extraplastid is explained in Fig. 1f. Plots for lateral and longitudinal diameters of a plastid (b) and an extraplastid (c). Areas and diameters were measured for 30 cells in each culture time. **Figure S2** Changes in expression level of genes involved in central carbon metabolism in the flat-plate culture. The transcript level of each gene was measured by quantitative RT-PCR, and corrected using transcript levels of 18S rRNA gene as an internal standard, and then normalized to the transcript level of each gene at 0 h. Genes are classified into six classes with respect to the peak of transcript level; genes of <2-fold change (a), 2 to 4-fold changes (b), 4 to 8-fold changes (c), 8 to 16-fold changes (d), 16 to 32-fold changes (e), and >32-fold change (f). Each value is an average ± standard deviation of three independent assays. **Figure S3** Culture under the darkness with addition of organic substances. *C. merolae* was cultured in a flask with rotary-shaking under the dark condition with addition of 200 mM glucose, 200 mM glycerol, 200 mM succinic acid, or 5 mM L-lactic acid for four weeks and OD_750_ was measured every one week. In the assay, the cells were exposed to room light at the sampling time for a short period of time.

## Abbreviations

2OG: 2-oxoglutarate
DHAP: dihydroxyacetone phosphate
G3P: glycerol 3-phosphate
G6P: glucose 6-phosphate
GAP: glyceraldehyde 3-phosphate
GAPC: glyceraldehyde 3-phosphate dehydrogenase
GK: glycerol kinase
GPD: glycerol 3-phosphate dehydrogenase
HA: hemagglutinin
LDH: lactate dehydrogenase
PEG: polyethylene glycol
PEP: phosphoenolpyruvate
PFK: ATP-dependent phosphofructokinase
PFP: PPi-dependent phosphofructokinase
PGAM: phosphoglycerate mutase
PGK: phosphoglycerate kinase
PGL: 6-phosphogluconolactonase
PK: pyruvate kinase
PYR: pyruvate
RPI: ribose-5-phosphate isomerase
TPI: triosephosphate isomerase
