# Supplementary material for: Activation of oxidative carbon metabolism by nutritional enrichment by photosynthesis and exogenous organic compounds in the red alga Cyanidioschyzon merolae: evidence for heterotrophic growth
Source: Springerplus. 2015 Sep 28;4:559. doi: 10.1186/s40064-015-1365-0 (PMC4586181; doi:10.1186/s40064-015-1365-0)
Supplement: Supplementary file 1 — Additional file 1: Table S1. List of primers for quantitative RT-PCR. All primers were designed by Primer Express software (Applied Biosystems). Slope and amplification efficiency (E) of each primer pair in quantitative-PCR are also shown. Figure S1 Changes in cell size and shape of C. merolae cell in the flat-plate culture. (a) A plot for areas of a plastid and an extraplastid at 0, 1, 3, and 6 h in the culture. Compartmentation of the plastid and extraplastid is explained in Fig. 1f. Plots for lateral and longitudinal diameters of a plastid (b) and an extraplastid (c). Areas and diameters were measured for 30 cells in each culture time. Figure S2 Changes in expression level of genes involved in central carbon metabolism in the flat-plate culture. The transcript level of each gene was measured by quantitative RT-PCR, and corrected using transcript levels of 18S rRNA gene as an internal standard, and then normalized to the transcript level of each gene at 0 h. Genes are classified into six classes with respect to the peak of transcript level; genes of <2-fold change (a), 2 to 4-fold changes (b), 4 to 8-fold changes (c), 8 to 16-fold changes (d), 16 to 32-fold changes (e), and >32-fold change (f). Each value is an average ± standard deviation of three independent assays. Figure S3 Culture under the darkness with addition of organic substances. C. merolae was cultured in a flask with rotary-shaking under the dark condition with addition of 200 mM glucose, 200 mM glycerol, 200 mM succinic acid, or 5 mM L-lactic acid for four weeks and OD750 was measured every one week. In the assay, the cells were exposed to room light at the sampling time for a short period of time. [file 40064_2015_1365_MOESM1_ESM.docx]

### Table S1. List of primers for quantitative RT-PCR. All primers were designed by Primer Express software (Applied Biosystems). Slope and amplification efficiency (*E*) of each primer pair in quantitative-PCR are also shown

| Primer name | Sequence (5' to 3') | Slope | *E*^a^ |
| --- | --- | --- | --- |
| CMA030C-F | CGTGTCCGCAGTCGTTCA | -3.66 | 0.88 |
| CMA030C-R | GGCGGTCGCGTCTTGA |  |  |
| CMA040C-F | GCGAACGGCGCATCAG | -3.61 | 0.89 |
| CMA040C-R | GGTCTTCCAGTGCTCGCTTCT |  |  |
| CMA145C/CMC188C/CMI306C/CMJ002C/CMK006C-F | CCCGGAGAAAGTCGTCTTGA | -3.39 | 0.97 |
| CMA145C/CMC188C/CMI306C/CMJ002C/CMK006C-R | GCGGAATAATGCCTTTGAACA |  |  |
| CMC021C-F | AGGGTGTCGCGGTGAAAG | -3.26 | 1.03 |
| CMC021C-R | CCGCATAGCTGACACCGACTA |  |  |
| CMC120C-F | GGGCACACGGCCTCTCT | -3.47 | 0.94 |
| CMC120C-R | TTGCCTCGGGTGCAGATC |  |  |
| CMD041C-F | TTCAACCCACGCGATGTG | -3.36 | 0.98 |
| CMD041C-R | AGAGTGCATAACCTGCACCAACT |  |  |
| CMD058C-F | CTGCGCGATCTCCGAAAC | -3.59 | 0.90 |
| CMD058C-R | GCCGATTTTAATGATGTCCTCAA |  |  |
| CMD113C-F | TTGCTCTTGGAGCCGGTTT | -3.47 | 0.94 |
| CMD113C-R | TGCCGCCTTCGTGTTGTT |  |  |
| CME095C-F | GCTCGCAAAGGCCGATAC | -3.61 | 0.89 |
| CME095C-R | GTCGGGATCCGGAACCA |  |  |
| CME145C-F | CACGCTACGGTGCTATTTCACA | -3.39 | 0.97 |
| CME145C-R | TGAGGACTTCGGGCTCGAT |  |  |
| CMF068C-F | TCACCACGGACCCAAAGC | -3.43 | 0.96 |
| CMF068C-R | CCGACACATTTCGCCACAT |  |  |
| CMF117C-F | TGCTTGGATGATTGGCATAATC | -3.68 | 0.87 |
| CMF117C-R | TCCAAAGCCGTGATGTTATCCT |  |  |
| CMH052C-F | CGTTGTTTCCCCACGTTCA | -3.49 | 0.93 |
| CMH052C-R | TTGGCTTCTCACCGTCATGA |  |  |
| CMH132C-F | ACGGCTTGAACGCACACA | -3.71 | 0.86 |
| CMH132C-R | TGGAGAATACCCGGGCAAT |  |  |
| CMI049C-F | TGGCATGACGGAGGAAGAG | -3.41 | 0.97 |
| CMI049C-R | TTGCGTGGACGGCTGTT |  |  |
| CMI084C-F | AAGCCGCTATCGATGAAATCC | -3.71 | 0.86 |
| CMI084C-R | TCGTTCCCGAATTCACTGTAGA |  |  |
| CMI162C-F | TTGTCCCCACGAGAAAACCT | -3.49 | 0.94 |
| CMI162C-R | TGCGGGACGCCAAACTT |  |  |
| CMI196C-F | CGGCAAAGCTTCGTGTACTCT | -3.63 | 0.89 |
| CMI196C-R | CGGCGCGCTCGATTAG |  |  |
| CMI224C-F | CATCAGCGACGCCCAGAT | -3.52 | 0.92 |
| CMI224C-R | TTTCAGGGCAGGGTCGAA |  |  |
| CMI273C-F | TTCCCGTGGGCACCTTAA | -3.71 | 0.86 |
| CMI273C-R | GCAACGCGGATACGTGTTC |  |  |
| CMJ042C-F | GGAATTCGGCATCGTTTCC | -3.32 | 1.00 |
| CMJ042C-R | GACGCTGGTCCCCGGTAT |  |  |
| CMJ051C-F | CAGGCGAATAACGTTTATTGCTT | -3.76 | 0.85 |
| CMJ051C-R | GCACCCGCCAGATGGA |  |  |
| CMJ055C-F | GCCGAGGGTGACAACGTT | -3.58 | 0.90 |
| CMJ055C-R | TGTGCGCCCACCTCAAC |  |  |
| CMJ173C-F | AATGCTCGTCGCTGTTTGG | -3.40 | 0.97 |
| CMJ173C-R | GGCAGGCTTTATGCCTTTCTG |  |  |
| CMJ250C-F | CTACCCGAAGCTTGACGGTAA | -3.38 | 0.98 |
| CMJ250C-R | GATACAAGGGCCACCGGTATC |  |  |
| CMJ272C-F | CGTTGTCCAGGTTCCAACGT | -3.57 | 0.90 |
| CMJ272C-R | CGTGTGCGCTTTCGTATGC |  |  |
| CMJ293C-F | GCGCGCTCTCGACATTCT | -3.63 | 0.89 |
| CMJ293C-R | CACGCTGTCGAGCAGTTCAG |  |  |
| CMJ305C-F | CGGACAAGGTATCGCATATCTCA | -3.54 | 0.92 |
| CMJ305C-R | CCCGCCCCTCGATTAACT |  |  |
| CMK041C-F | CGGCAAGTCGGCGTCTT | -3.56 | 0.91 |
| CMK041C-R | CGACGCCAAAACGAATGTC |  |  |
| CMK131C-F | CGATCTCCTGGTTACGAATATCG | -3.51 | 0.93 |
| CMK131C-R | CGTTGCAGGCGCGTTT |  |  |
| CMK188C-F | TGTCAAGAGCTCGGCTACGTACT | -3.29 | 1.01 |
| CMK188C-R | GAGCATGCATTCCGCGTTA |  |  |
| CML036C/CML059C/CMM231C-F | GCAGGTCGCGCAATATCG | -3.46 | 0.95 |
| CML036C/CML059C/CMM231C-R | GTGCAAAGTTCTGACCCATGAC |  |  |
| CML209C-F | ACGATGGCCAGATGGATGA | -3.55 | 0.91 |
| CML209C-R | TAGCGCCGCCGTCACT |  |  |
| CMM068C-F | CATCAGCGGTGTCGGAATG | -3.51 | 0.93 |
| CMM068C-R | TCCTGAGGTACGCGTGCAT |  |  |
| CMM167C-F | TCGAGTCCACTGGCGTGTT | -3.31 | 1.00 |
| CMM167C-R | CGCACCGCCTTTCAGATG |  |  |
| CMM196C-F | TGCCGGCAAGTCAAATACC | -3.65 | 0.88 |
| CMM196C-R | GCCCACGGAACGAAACTTT |  |  |
| CMM299C-F | TGTAGCCCTGCCCATGCT | -3.60 | 0.89 |
| CMM299C-R | AGCGCTCCTGTCGACGAT |  |  |
| CMN017C-F | CACTTTCGGAGGTGCTTTGC | -3.71 | 0.86 |
| CMN017C-R | CCGGCATACGGAGCTTGAT |  |  |
| CMN233C-F | TCATTGCGTCGCCGTATG | -3.53 | 0.92 |
| CMN233C-R | GCAGGGTACGCAGGTCAATT |  |  |
| CMN285C-F | CTCGTATGCGATGCTTTTGG | -3.43 | 0.96 |
| CMN285C-R | GCAGCCTGGAGTGGTGTCA |  |  |
| CMO121C-F | TGCGCGGAGCGTACGT | -3.67 | 0.87 |
| CMO121C-R | GGATCACATCCGGGTGCTT |  |  |
| CMO124C-F | TGCCGATGGTGGAGAAGTTC | -3.59 | 0.90 |
| CMO124C-R | CCCGACCCAGTCCCAAA |  |  |
| CMO128C-F | TCAAGGATTGTGCGGCAAA | -3.61 | 0.89 |
| CMO128C-R | GCATTGCGCCGGAGACT |  |  |
| CMO229C-F | CCGTCGTTGCTCTCTTGTGA | -3.75 | 0.85 |
| CMO229C-R | GTCGAGCACGCGTTTCG |  |  |
| CMO245C-F | CCCGAGGACGCGGATATAA | -3.58 | 0.90 |
| CMO245C-R | TCGCACCCGGTTGCA |  |  |
| CMO276C-F | CCCCGACGCTGGTATGAG | -3.76 | 0.85 |
| CMO276C-R | AAACAATCCGCTCGGTATGC |  |  |
| CMO291C-F | CCATAGACGGAGCGGATGAG | -3.47 | 0.94 |
| CMO291C-R | GCACCACCGCGTCCTTT |  |  |
| CMP129C-F | GAAGCACAGGGCGACTTGAC | -3.60 | 0.90 |
| CMP129C-R | TCGATGACCTTACAGGCAAGCT |  |  |
| CMP193C-F | GTGCGTTTCCGCTGCTAAA | -3.64 | 0.88 |
| CMP193C-R | GCATCCGCAAAGCCCTTAT |  |  |
| CMP260C-F | CGACGGCACCGATTGC | -3.83 | 0.82 |
| CMP260C-R | TGCACCGGATAGAGACCTTTG |  |  |
| CMQ172C-F | GCCATCGGTTTATGCCATGT | -3.49 | 0.93 |
| CMQ172C-R | TGCAGTGCTACCCCGAAATC |  |  |
| CMQ191C-F | GGGAACGATTCGGTGAAGAA | -3.72 | 0.86 |
| CMQ191C-R | ATCCCACCGTAGGCCTGATT |  |  |
| CMQ234C-F | GGCTGCGTTCCGTCAAAA | -3.66 | 0.88 |
| CMQ234C-R | TTCGAAGCTCCCGGACTTT |  |  |
| CMR014C-F | CCGCGACATCATGCAGAAT | -3.57 | 0.91 |
| CMR014C-R | CCGGCTGTTCCATCGTAAAG |  |  |
| CMR476C-F | GGCTGCTGGTTATGTGTTCATG | -3.45 | 0.95 |
| CMR476C-R | CGCTCTCCACGAGAAAAGCT |  |  |
| CMS195C-F | TCGCCGCATGGAATGAA | -3.62 | 0.89 |
| CMS195C-R | TTGAGGATGTTGGCCGTTATC |  |  |
| CMS272C-F | ATTCCGAGCAATGTTCTGGATT | -3.39 | 0.97 |
| CMS272C-R | CAGCGGCCCCTTGATG |  |  |
| CMS327C-F | AGAAGACGCCAAGGGACTTCT | -3.78 | 0.84 |
| CMS327C-R | TTCAAGCACACAAACGGGATT |  |  |
| CMT034C-F | GGCAGCAACCAAGGAGGAA | -3.67 | 0.87 |
| CMT034C-R | TCGCGCTGCGCTTGA |  |  |
| CMT209C-F | TTGTGGATGCAGCAAAACGT | -3.62 | 0.89 |
| CMT209C-R | GCCTTCGAGCCGAACAATC |  |  |
| CMT216C-F | CGGACGACGGCGTTACC | -3.46 | 0.94 |
| CMT216C-R | TTAACCGCTGCGTGATGCT |  |  |
| CMT256C-F | AGGCCCGAGCGGAGAA | -3.33 | 1.00 |
| CMT256C-R | CAGTGCGAACGGTGAAAGTTC |  |  |
| CMT285C-F | GCGATTATGCCGTGCTTGA | -3.62 | 0.89 |
| CMT285C-R | CGTAAAATCATCGGCAACGA |  |  |
| CMT362C-F | GCACCGGATGTCTTCCAAA | -3.54 | 0.92 |
| CMT362C-R | CCGGAACACTCGGATTACAGA |  |  |
| CMT412C-F | TTGTTGATGCGACCTGTATGC | -3.44 | 0.95 |
| CMT412C-R | GGTTCGGCAACAGTACAACGT |  |  |
| CMT497C-F | CGGCCTTAACCCGGAAA | -3.55 | 0.91 |
| CMT497C-R | CGCGGTCGTGAAGGTTTT |  |  |
| CMT561C-F | GCATGCGGGCCCTGTA | -3.54 | 0.92 |
| CMT561C-R | TGTTTTTGACGCCCTTTGGT |  |  |
| CMT582C-F | CGTCCTTCCGACGGTTCA | -3.60 | 0.90 |
| CMT582C-R | GGACTTCACCTCGCCAGTTG |  |  |
| CMT611C-F | GGCCATCGGCGACCAT | -3.39 | 0.97 |
| CMT611C-R | CGATCTCGTTTTCCGTTTGG |  |  |
| CMT633C-F | CGCATGGGTGTGGAAAGG | -3.69 | 0.87 |
| CMT633C-R | ATGGAAGGTGCAACCACAACT |  |  |
| CMV013C-F | GCATCTGGCGAAGTCAAAGG | -3.85 | 0.82 |
| CMV013C-R | CGCAAATTCTGCTCTCTTATAACATT |  |  |
| CMV014C-F | TGGCATTCAACTCGACTAAAGG | -3.44 | 0.95 |
| CMV014C-R | TTCGTTTGCAGGTCGATTCA |  |  |
| CMV153C-F | ATGGCCGAATTATTCGGAAA | -3.83 | 0.82 |
| CMV153C-R | GGTGCTGAAAATAAATGCATCGA |  |  |
| CMV154C-F | TGCAGGCATGTTGCATTATACA | -3.24 | 1.04 |
| CMV154C-R | TCCGGGACCTCGAATCACTA |  |  |
| CMW001C-F | TGTTTTTCTTTAAATTTTATTCGCCTACT | -3.46 | 0.94 |
| CMW001C-R | CTTTATTAAATAACCATAAAAAACTTACACACATT |  |  |
| CMW002C-F | TTGGTGGAATGGTGATCGTTATT | -3.58 | 0.90 |
| CMW002C-R | GCGCTGTCACGACTATCTATAATCC |  |  |

^a^Amplification efficiency (*E)* for each gene was determined from following equation: *E* = 10^-1/Slope^ -1.


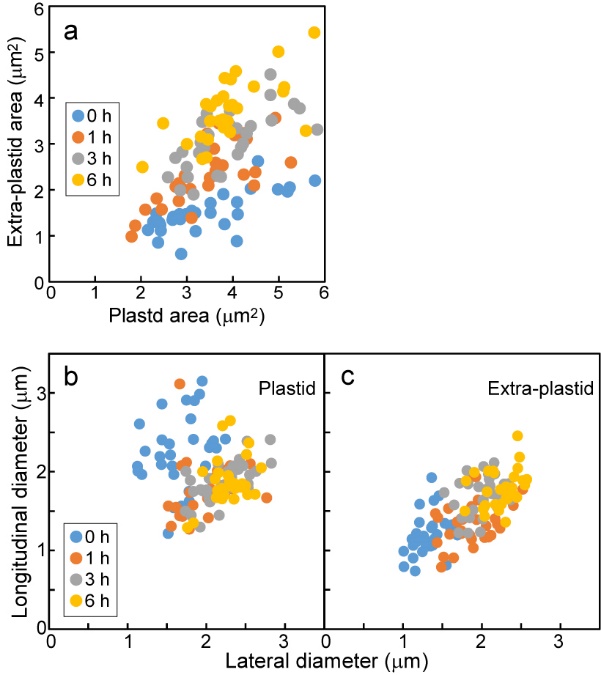


### Figure S1 Changes in cell size and shape of *C. merolae* cell in the flat-plate culture. (a) A plot for areas of a plastid and an extraplastid at 0, 1, 3, and 6 h in the culture. Compartmentation of the plastid and extraplastid is explained in Figure 1f. Plots for lateral and longitudinal diameters of a plastid (b) and an extraplastid (c). Areas and diameters were measured for 30 cells in each culture time


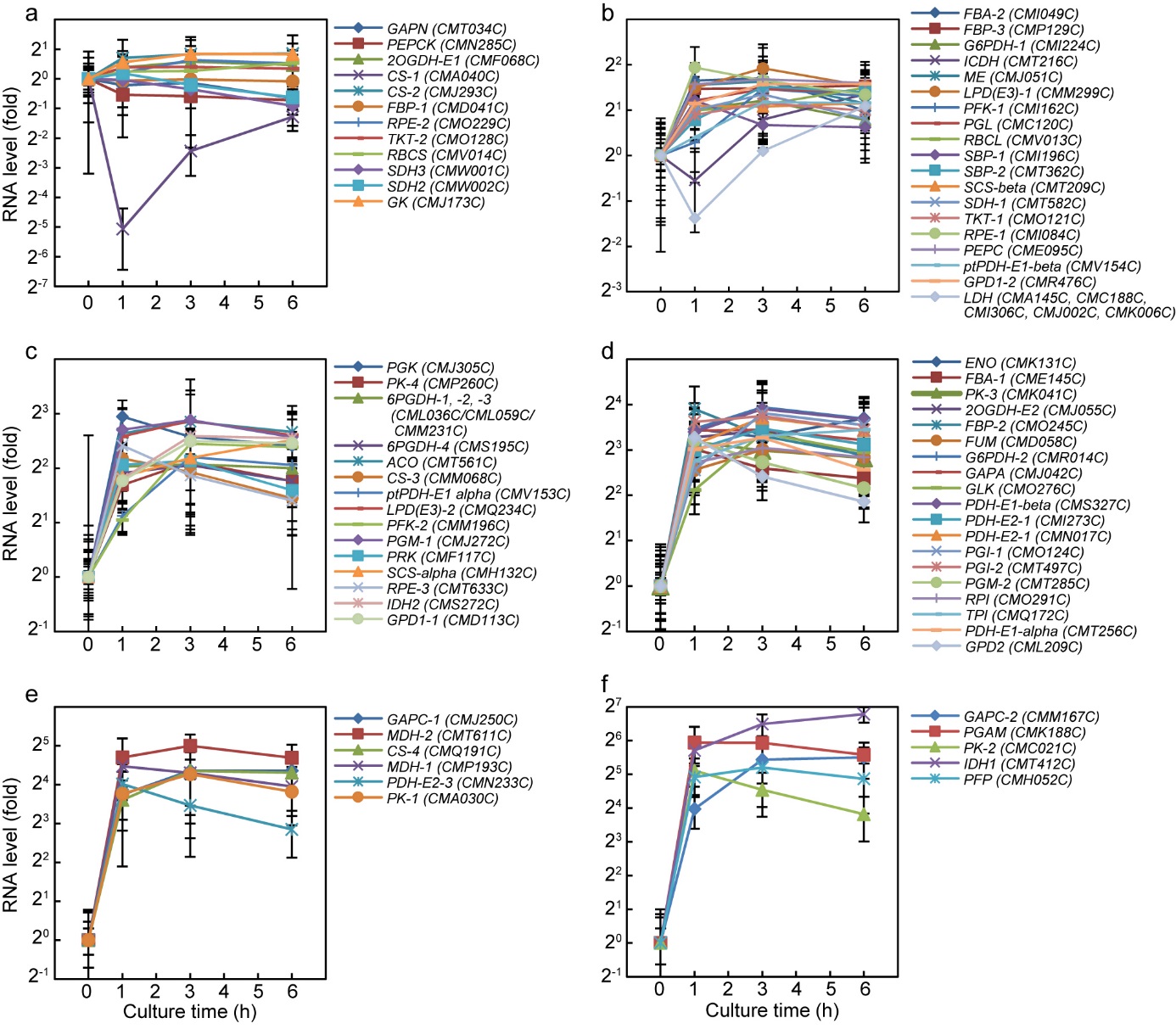


### Figure S2 Changes in expression level of genes involved in central carbon metabolism in the flat-plate culture. The transcript level of each gene was measured by quantitative RT-PCR, and corrected using transcript levels of 18S rRNA gene as an internal standard, and then normalized to the transcript level of each gene at 0 h. Genes are classified into six classes with respect to the peak of transcript level; genes of <2-fold change (a), 2 to 4-fold changes (b), 4 to 8-fold changes (c), 8 to 16-fold changes (d), 16 to 32-fold changes (e), and >32-fold change (f). Each value is an average ± standard deviation of three independent assays


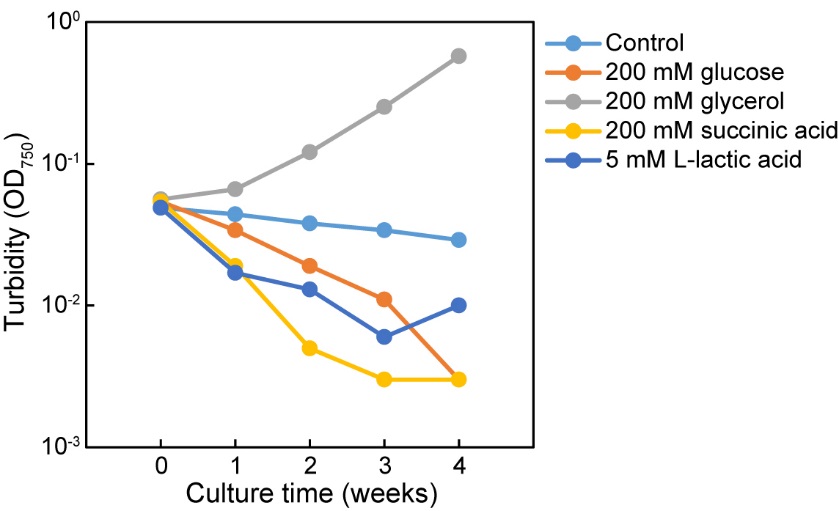


### Figure S3 Culture under the darkness with addition of organic substances. *C. merolae* was cultured in a flask with rotary-shaking under the dark condition with addition of 200 mM glucose, 200 mM glycerol, 200 mM succinic acid, or 5 mM l-lactic acid for four weeks and OD_750_ was measured every one week. In the assay, the cells were exposed to room light at the sampling time for a short period of time.
